# Melanopsin-mediated pupillary responses in bipolar disorder—a cross-sectional pupillometric investigation

**DOI:** 10.1186/s40345-020-00211-3

**Published:** 2021-03-01

**Authors:** Helle Østergaard Madsen, Shakoor Ba-Ali, Steffen Heegaard, Ida Hageman, Ulla Knorr, Henrik Lund-Andersen, Klaus Martiny, Lars Vedel Kessing

**Affiliations:** 1 Copenhagen Affective Disorder Research Center (CADIC), Mental Health Center Copenhagen, Rigshospitalet, Edel Sauntes Allé 10, 2100 Copenhagen Ø, Denmark; 2 Department of Ophthalmology, Rigshospitalet, Glostrup, Denmark; 3 Mental Health Services, Capital Region of Denmark, Copenhagen, Denmark

**Keywords:** Bipolar disorder, Mood disorders, Melanopsin, Pupillary reflex, Pupillometry, Circadian rhythm, Retina

## Abstract

**Background:**

Visible light, predominantly in the blue range, affects mood and circadian rhythm partly by activation of the melanopsin-containing intrinsically photosensitive retinal ganglion cells (ipRGCs). The light-induced responses of these ganglion cells can be evaluated by pupillometry. The study aimed to assess the blue light induced pupil constriction in patients with bipolar disorder (BD).

**Methods:**

We investigated the pupillary responses to blue light by chromatic pupillometry in 31 patients with newly diagnosed bipolar disorder, 22 of their unaffected relatives and 35 healthy controls. Mood state was evaluated by interview-based ratings of depressive symptoms (Hamilton Depression Rating Scale) and (hypo-)manic symptoms (Young Mania Rating Scale).

**Results:**

The ipRGC-mediated pupillary responses did not differ across the three groups, but subgroup analyses showed that patients in remission had reduced ipRGC-mediated responses compared with controls (9%, *p* = 0.04). Longer illness duration was associated with more pronounced ipRGC-responses (7% increase/10-year illness duration, *p* = 0.02).

**Conclusions:**

The ipRGC-mediated pupil response to blue light was reduced in euthymic patients compared with controls and increased with longer disease duration. Longitudinal studies are needed to corroborate these potential associations with illness state and/or progression.

## Introduction

In bipolar disorder (BD), a multitude of external factors can interfere with the course of disease and provoke the onset of an affective episode, i.e. depression or mania (Young and Dulcis 2015). Contributing factors include disturbances in biological rhythms caused by e.g. sleep deprivation or change of season. As such, there is evidence for a distinct seasonal pattern in health care utilization in BD with a peak rate of admissions for mania in spring/summer and for depression in winter (Geoffroy et al. 2014) just as patients with BD report increased fluctuations in mood and behavior in relation to change of seasons (Geoffroy et al. 2014). The main driver of this seasonal variation is presumably the change in daylight (Geoffroy et al. 2014; Aguglia et al. 2017). In line with this, chronotherapeutic modalities such as light and dark therapy can be used adjunctively in the treatment of depression and mania, respectively (Gottlieb et al. 2019; van Houl et al. 2020). A hypothesis of so-called retinal super-sensitivity to light has been suggested in BD (Lewy et al. 1985, 1981). Early support for this hypothesis included the findings of increased melatonin suppression in response to light in individuals with BD as well as in offspring of patients with BD (Lewy et al. 1985, 1981; Nurnberger et al. 1988). Other studies could not corroborate these findings (Lam et al. 1990; Whalley et al. 1991; Ritter et al. 2019). Aberrant melatonin suppression in patients and offspring could indicate that aberrant light sensitivity constitutes an inherited trait biomarker. Such endophenotypes can potentially inform on underlying biological vulnerability or predict illness emergence in un-affected carriers of the trait. State-dependent biomarkers reflect on the phenotypical presentation of current illness states and can potentially aid in diagnostic and staging processes.

As light reaches the retina, specialized neurons—rods, cones and intrinsically photosensitive retinal ganglion cells (ipRGCs)—perceive the signal (Hattar et al. 2002). The ipRGCs play essential roles in the transmission of light for non-visual purposes such as the pupillary light reflex, suppression of melatonin and regulation of diurnal rhythm, alertness and mood (Lazzerini Ospri et al. 2017; Fernandez et al. 2018). The ipRGCs are predominantly sensitive to high-intensity short wavelength—blue—light (Hattar et al. 2002; Park et al. 2011). IpRGC-activation by blue light elicits the so-called post-illumination pupillary response (PIPR),i.e. a pupillary constriction that persists past the termination of a light stimulus. The PIPR can be swiftly and non-invasively assessed with chromatic pupillometry (Park et al. 2011), i.e. recording and quantification of the pupillary responses to monochromatic light stimuli (Park et al. 2011). Pupillometric evaluation of the ipRGC-mediated responses may provide a potential alternative to the invasive, time consuming, and expensive assessment of circadian system light sensitivity by melatonin suppression experiments. The disadvantages of the method include numerous sources of intra- and inter-individual variation, such as circadian and seasonal timing, iris color, medication, etc. (Kelbsch et al. 2019).

There is some evidence that the PIPR is attenuated in persons with unipolar depression (Roecklein et al. 2013; Berman et al. 2018), but it has not been investigated in persons with BD. In persons without affective disorder, a more pronounced PIPR was associated with higher levels of self-reported lifetime hypomanic/manic-like experiences (Bullock et al. 2019). In this first pupillometric investigation in BD, we assessed trait and state biomarker characteristics of the ipRGC-mediated pupillary response in patients with newly-diagnosed BD and we explored potential associations with illness variables.

## Methods

In a cross-sectional design, we investigated the pupillary responses in patients with newly diagnosed BD, their unaffected first-degree relatives and a healthy control group between September 2018 and November 2019. All participants attend the research facility for regular follow-up visits as a part of the Bipolar Illness Onset (BIO)-study (Kessing et al. 2017). BIO-participants who had no prior or current eye disorder, eye trauma, eye surgery or family history of glaucoma were invited to participate in the pupillometric study.

The study was approved by the Committee on Health Research Ethics of the Capital region of Denmark (H-7-2014-007) and complied with the Declaration of Helsinki. Participants all provided written informed consent.

### Participants

#### Patients with bipolar disorder (BD)

BIO-participants with BD have been recruited among outpatients at the Copenhagen Affective Disorder Clinic with a recent ICD-10 diagnosis of BD or single manic episode (WHO 1992). The Copenhagen Affective Disorder Clinic offers a specialized two-year treatment program to all persons with newly diagnosed BD in the greater Copenhagen area. All referred patients undergo a diagnostic assessment by a psychiatrist. The ICD-10 diagnosis of BD is further specified as type I or II as by DSM-V criteria (American Psychiatric Association 2015).

#### Unaffected first-degree relatives (UR)

Siblings and children to the patients with BD have been included in the BIO-study. These unaffected relatives have had no personal history of substance abuse, psychotic illness or mood disorders according to the ICD-10 at the time of inclusion to the study.

#### Healthy control participants (HC)

Age-matched control individuals without any personal or family history of psychiatric disorders have been recruited to the BIO-project among blood donors at Rigshospitalet, Copenhagen.

### Assessments

#### Psychiatric evaluation

At inclusion to the BIO-study, all participants underwent a semi-structured diagnostic interview, Schedules in Clinical Assessment in Neuropsychiatry (SCAN), to confirm the clinical diagnosis of BD and to exclude current or prior psychopathology in the UR and HC groups. The interview and clinical evaluation were carried out by trained PhD students (MDs or MSc in psychology). For BD participants, disease duration was recorded as years since their first depressive or (hypo-)manic episode. The severity of current affective symptoms was assessed by interview-based clinical rating scales, i.e. the Hamilton Depression Rating Scale – 17 item version (HDRS) and the Young Mania Rating Scale (YMRS). We used the mood scores to classify the current affective state as follows: remission (both scores ≤ 7), depression (HDRS ≥ 13 and YMRS < 13) and hypomania/mixed state (YMRS ≥ 13) (Kessing et al. 2017; Faurholt-Jepsen et al. 2016). The participants’ habitual time of sleep onset and wake-up time within the last 30 days were assessed by the Pittsburgh Sleep Quality Index (PSQI). The time of assessment was defined as the time from habitual wake up time to the recorded time of pupillometry. Current medication and length of day of assessment (http://www.dagenslaengde.dk) was recorded.

#### Baseline eye examination

We used a Snellen chart to confirm a best corrected visual acuity within normal limits (≥ 0.8). Retinal condition was assessed with optical coherence tomography (OCT), using spectral domain OCT (Heidelberg SD-OCT; Heidelberg Engineering, Heidelberg, Germany). OCT is non-invasive imaging technique that provides high resolution images of the retina through scattering of long-wavelength light. The protocol included macular and peripapillary imaging. Iris color was recorded as dark (brown, black) or light (blue, green, grey).

#### Pupillometry

We used an automated binocular pupillometer (DP-2000 Human Laboratory Pupillometer, NeurOptics, CA, USA) to elicit monocular monochromatic light stimuli under simultaneous tracking and recording of both pupillary diameters. The light was presented through a diffusing screen (50° × 35° of visual angle) at an illumination level of 100 lux. For the red stimulus with peak wavelength and bandwidth of 633 nm (17 nm), this corresponded to 15.23 log quanta/cm2/second. For the blue stimulus of 463 nm (25 nm), it corresponded to 15.27 log quanta/cm2/second. The study eye was arbitrarily chosen or if there was a difference in visual acuity, we used the better eye. Prior to examination, the study eye was dilated with mydriatic eyedrops (10% phenylephrine hydrochloride and 1% tropicamide) to ensure a pupil diameter > 5 mm before stimulation in order to induce a maximal melanopsin-mediated pupillary response (Ba-Ali et al. 2018). The protocol consisted of 5 min dark adaptation, 10 s baseline recording, 20 s stimulation of the dilated eye with red light and 60 s recording of the pupillary diameters after end of stimulation. After a 5 min break, the protocol was repeated using blue light. Assessments were performed during daytime (8.30 AM – 5 PM) to limit the diurnal variation in the pupillary dynamics.

The raw data on pupillary diameters from the consensual eye—i.e. the non-dilated eye—were imported into the R-statistical software package, version 3.5.1. We inspected a graphic depiction of the pupil diameter (mm) as a function of time (s) to assess the validity of the examination. Blink artefacts were removed with a mathematical algorithm or by manual linear interpolation. Pupillometry outcomes included the pupillary diameter at baseline, the maximal pupil contraction at time 13–15 (3–5 s within the stimulation) and the post-illumination pupil contraction measured at time 40–60 s ( 10–30 s post-illumination). Mean baseline diameters for the initial 10 s prior to stimulation are reported in millimeter. For the two contraction parameters, the outcomes are reported as the difference between 1 and the mean pupillary diameter for a specified timeframe (see definitions below) divided by the baseline diameter. This normalization to baseline was performed to adjust for the inter-individual differences in pupil size caused by differences in age, sympathetic tone or medication. By the subtraction from 1, the outcomes are interpreted as a degree of contraction, i.e. a higher value reflects a higher contractile res*p*onse corresponding to a smaller pupil diameter.

The pupillometric outcomes:

Baseline pupil diameter (PD_baseline_): mean pre-stimulus pupil diameter (mm) from time 0–10 s.

Maximal contraction (C_max_): 1 – (mean pupil diameter (mm) during stimulation from time 13–15 s / PD_baseline_ (mm)).

Post-illumination pupillary response (PIPR_late_): 1 – (mean pupil diameter (mm) from time 40–60 s / PD_baseline_ (mm)).

The C_max_ is a product of joined rod and cone activation. The PIPR_late_ is a product of ipRGC-activation (Ba-Ali et al. 2018).

### Statistical analysis

Data were analyzed in the R-statistical software package, version 3.5.1. We report continuous variables as mean and standard deviation (SD) or median and interquartile range (IQR) according to their distribution. Categorical data are presented as numbers and proportions. Comparisons of baseline data were performed in the *tableone* package using Kruskal–Wallis, ANOVA or Fisher’s *exact-*test according to the distributions. Spearman correlation tests were performed with the *cor.test* function of the *stats* package.

The a priori analysis plan had the main objective to compare the PIPR_late_ across the 3 groups. Comparisons were performed in a linear mixed effect model (*lme* function in the *nlme* package) to account for the familial relationship between some participants. Estimates of the mean PIPR_late_ and the 95% confidence intervals (CI) for each group were obtained by an initial unadjusted model with the PIPR_late_ as the dependent variable, family relation as a random effect and group as a fixed effect. The model was then adjusted for the covariates of age and sex and lastly a fully adjusted model also included the daylength on time of assessment. To avoid a potential effect of the current affective state in BD, we ran the same models including only patients in remission.

With the secondary objective to evaluate state-dependent variation in the PIPR_late_, we tested the effects of affective scores (HDRS and YMRS) and of current affective state (remission, depression, hypomania/mixed state) on the PIPR_late_ in patients with BD. This was done in separate linear regression models adjusted for sex and age using the *lm* function of the *stats* package. Model fit was assessed by visual inspection of the relevant residual plots.

Thirdly, we investigated associations between the PIPR_late_ and illness variables: psychotropic medication (number of agents (0, 1, ≥ 2) and single agents (lithium, lamotrigine, antidepressants, antipsychotics), BD type (I or II) and illness duration (years since first depressive or (hypo-) manic episode) in separate linear regression models (1:unadjusted, 2: age- and sex-adjusted) within the BD group.

## Results

### Descriptives

A total of 91 participants underwent pupillometry; 34 patients with BD, 22 UR and 35 HC. We excluded three BD participants due to invalid pupillometric assessments (blinking or deviation of the eye) which left 31 BD participants for analysis (Table 1). There were 4 pairs of relatives in the BD and UR groups, and one pair within the UR group. The median age of the BD and HC samples were 28–29 years and the UR group was slightly younger with a median age of 24 years (*p* = 0.008). For all participants, OCT scans and visual acuity assessments were normal. Pupillometry was performed at a similar median time of 12 noon, 11.45 AM and 12 noon in the BD, UR and HC groups, respectively (*p* = 0.39) and the groups did not differ in regard to time since habitual wake up time, *p* = 0.58 (Table 1). In the BD group, 9 persons were diagnosed with BD type I and 22 persons with BD type II. Median disease duration was 10 years, and 27 persons (77%) had been diagnosed with BD within the last three years. All patients were treated with psychotropic medication and the majority (n = 21, 67%) did not meet the criteria for a current affective episode but presented with some residual symptoms (HDRS and YMRS < 13).

**Table 1:** Characteristics of patients with bipolar disorder (BD), unaffected relatives (UR) and healthy controls (HC)

	BD	UR	HC	p^†^
n	31	22	35	
Age (median [IQR])	29 [25–32.5]	24 [22–28.5]	28 [25–31.5]	0.008
Male sex, n (%)	11 (35.5)	6 (27.3)	12 (34.3)	0.803
Dark eyes, n (%)	7 (22.6)	2 (9.1)	6 (17.1)	0.454
Time of assessment, minutes (mean (SD))	263 (143)	292 (154)	302 (150)	0.578
Daylength, minutes (median [IQR])	778 [685–943]	915 [813–955]	852 [703–972]	0.176
HDRS (median [IQR])	8 [5.5–13]	1 [0–2]	0 [0–1.5]	< 0.001
YMRS (median [IQR])	2 [0–7]	0 [0–1]	0 [0–0]	< 0.001
Bipolar type 2, n (%)	22 (71)			
Current phase, n (%)				
Remission	9 (29.0)			
Depression	7 (22.6)			
Hypomania/mixed	3 (9.7)			
Lithium, n (%)	12 (38.7)			
Lamotrigine, n (%)	18 (58.1)			
Antipsychotic, n (%)	15 (48.4)			
Antidepressant, n (%)	6 (19.4)			
Number of agents, n (%)				
0	0 (0)			
1	10 (32.3)			
2 or more	21 (67.7)			

Time of assessment: minutes since habitual wakeup time; Daylength: daylight minutes on day of assessment. *HDRS* hamilton depression rating scale, 17 item version. *YMRS* young mania rating scale. Current phase: remission (HDRS and YMRS < 8), depression (HDRS >/= 13 and YMRS < 13), hypomania/mixed (YMRS >/= 13). †Fishers exact test for categorical variables, *t*-test/Kruskal–Wallis for continuous variables

There was no correlation between age and the PIPR_late_ across the total sample (r = 0.15, p = 0.17) nor within HC (r = − 0.11, p = 0.52) or UR (r = 0.29, p = 0.19) subgroups. In the BD group, there was a trend toward increasing PIPR with increasing age (r = 0.34, p = 0.06).

### The PIPR_late_ as a trait vulnerability marker in BD

**Fig. 1:**
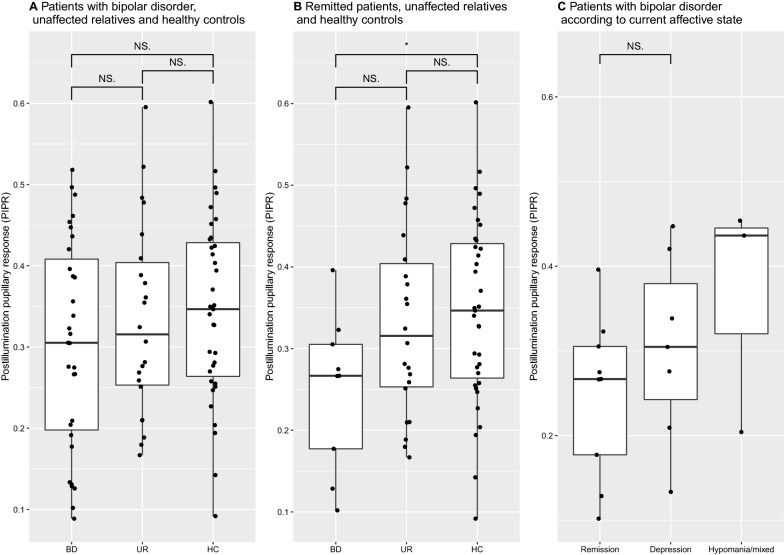
The post-illumination pupillary response to blue light (PIPR) according to group and affective state. *BD* bipolar disorder, *UR* unaffected relative, *HC* healthy control individual. *NS* nonsignificant comparison. Panel A includes 31 persons with BD, 22 UR and 35 HC. Panel B includes 9 persons with BD in remission, 22 UR and 35 HC. Panel C includes 9 persons with BD in remission, 7 persons in depression and 3 persons in hypomanic/mixed state. *denotes a *p*-value < 0.05

The blue induced PIPR_late_ did not differ significantly between the BD, UR and HC groups, nor with adjustment for age-, sex- and photoperiod (Fig. 1a, Table 2). The PIPR_late_ was reduced in the 9 BD participants in current remission (PIPR_late_ = 0.26 (95% CI: 0.20–0.33)) compared with the HC group (PIPR_late_ = 0.35 (CI: 0.31–0.38)), p = 0.04. but not with the UR group, PIPR_late_ = 0.33 (CI: 0.28–0.38), p = 0.34 (Fig. 1, Table 2).

**Table 2:** Pupillary outcomes in persons with bipolar disorder (BD), unaffected relatives (UR) and healthy controls (HC)

	BD (n = 31)		UR (n = 22)		*p*	HC (n = 35)		*p*	*p*
	Mean	95% CI	Mean	95% CI	BD-UR	Mean	95% CI	BD-HC	UR-HC
Blue light									
PIPR_late_	0.31	0.27–0.35	0.34	0.29–0.39	0.29	0.35	0.31–0.39	0.20	0.87
PD_baseline_ (mm)	7.09	6.81–7.40	7.53	7.17–7.89	0.13	7.32	7.04–7.60	0.28	0.35
C_max_	0.62	0.60–0.64	0.63	0.61–0.65	0.50	0.62	0.60–0.64	0.80	0.29
Red light									
PD_baseline_ (mm)	7.10	6.81–7.38	7.57	7.23–7.90	0.10	7.31	7.03–7.58	0.28	0.22
C_max_	0.49	0.46–0.52	0.48	0.45–0.51	0.56	0.51	0.48–0.54	0.33	0.14

	Comparisons of UR and HC with BD in current remission								
	Remission (n = 9)								
Blue light									
PIPR_late_	0.26	0.20–0.33	0.33	0.28–0.38	0.34	0.35	0.31–0.38	0.04	0.67
PD_baseline_ (mm)	7.04	6.49–7.61	7.53	7.17–7.89	0.38	7.32	7.04–7.60	0.38	0.36
C_max_	0.60	0.56–0.64	0.63	0.61–0.65	0.15	0.62	0.60–0.64	0.36	0.35
Red light									
PD_baseline_ (mm)	7.08	6.55–7.60	7.57	7.23–7.91	0.36	7.31	7.03–7.58	0.44	0.23
C_max_	0.44	0.39–0.49	0.48	0.44–0.51	0.42	0.51	0.48–0.54	0.02	0.13

*PIPR*_*late*_ The mean post-illumination pupillary contraction measured at 10–30 s after stimulation, *PD*_*baseline*_ mean pupil diameter during the 10 s before illumination; *C*_*max*_ mean pupillary contraction at 3–5 s during illumination. Remission is defined as Hamilton Depression Rating Scale and Young Mania Rating Scale < 8

The baseline pupil diameters measured prior to each stimulation did not differ significantly between the groups.

### The PIPR_late_ as a state-marker of BD

There were no significant associations between the PIPR_late_ and HDRS-score (β = 0.001, p = 0.77) or YMRS-score (β = 0.004, p = 0.35) in patients with BD. This did not change with adjustment for sex and age. There were no significant differences in the PIPR_late_ between BD-participants in different affective states: current remission (PIPR_late_ = 0.25 (95% CI = 0.17–0.32)), depression (PIPR_late_ = 0.30 (95% CI = 0.22–0.39)) or hypomania/mixed state (PIPR_late_ = 0.36 (95% CI = 0.23–0.50)), although the median values displayed a trend towards a more pronounced PIPR_late_ during both depressive and hypomanic/mixed states (Fig. 1c).

### Associations between the PIPR_late_ and illness variables in BD

**Fig. 2:**
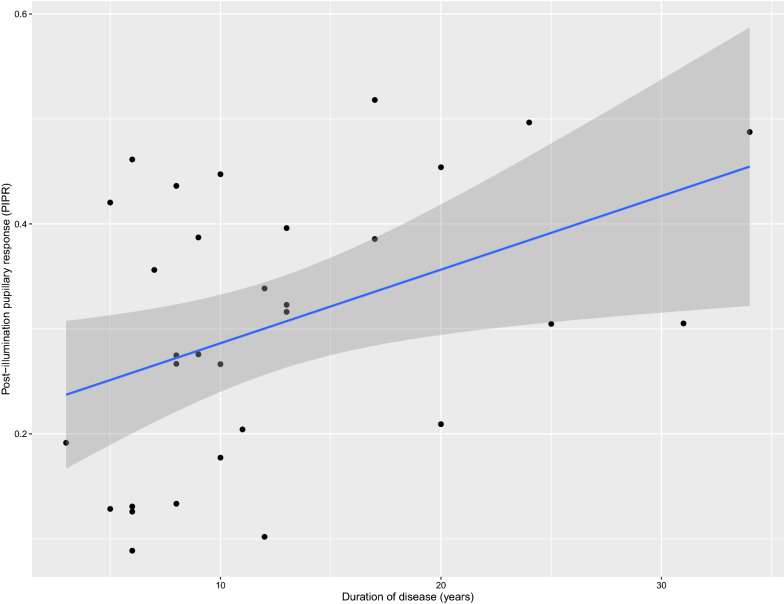
The post-illumination pupillary response to blue light (PIPR) as a function of illness duration (years since initial depression/mania/hypomania. A higher value indicates a greater post-illumination contraction relative to baseline. The linear regression model predicts a 7% increase in the post-illumination pupillary response to blue light for every 10-year increase in disease duration, p = 0.02

In the BD group, the PIPR_late_ and illness duration was positively associated (β = 0.007, p = 0.02) corresponding to a 7% increase in the PIPR_late_ for every ten-year increase in illness duration (Fig. 2). This effect was significant with adjustment for sex, but not for sex and age (β = 0.007, *p* = 0.11).

There were no significant associations between the PIPR_late_ and BD type (*p* = 0.88), number of psychotropic agents (*p* = 0.69) or any single drug type or group (lithium (p = 0.25), lamotrigine (p = 0.93), antipsychotics (p = 0.83), antidepressants, * p* = 0.85)).

## Discussion

From this first pupillometric assessment of patients with BD, we report indications that the ipRGC-mediated pupillary responses are altered along with the duration of illness and potentially across different illness states. However, we do not see any indications of aberrant ipRGC-responses being an inherited trait phenomenon in BD.

Although the retinal super-sensitivity hypothesis initially received support from a few studies reporting increased melatonin suppression in patients with BD and in unaffected adolescents with a high-risk profile for BD (Lewy et al. 1985, 1981; Nurnberger et al. 1988; Nathan et al. 1999), later studies challenged the hypothesis (Lam et al. 1990; Whalley et al. 1991; Ritter et al. 2019). Increased melatonin suppression was not found in remitted, drug-free patients (Ritter et al. 2019) nor in a recent well-controlled study using blue light for specific activation of the ipRGC pathway in euthymic patients with BD-I (Ritter et al. 2019). The latter study further reported that the subjective alerting response to blue light was attenuated in patients with BD compared with controls. This is much in line with the group of remitted patients in our study who displayed attenuated ipRGC-responses compared with controls. This finding was, however, based on only 9 remitted patients why it should be interpreted with caution. However, from the larger BIO-cohort, our group has shown, that the BD-group has disordered sleep compared with unaffected relatives and healthy controls even in remitted phases (la Cour Karottki et al. 2020). This inter-episode sleep disturbances may be associated with aberrant ipRGC-transmission. In healthy adolescents and young adults, a more pronounced PIPR is associated with a propensity for delayed sleep timing (Meijden et al. 2016).

We were not able to identify differences in the PIPR across different affective states or associations with symptom severity. This may relate to either sample size (type II error) or the mild-to-moderate symptom load in the present population. The median scores do indicate a trend toward a more pronounced PIPR in depressive and especially in hypomanic/mixed state compared with remission. The prior pupillometric studies in unipolar depression have not assessed the associations between the PIPR and symptom severity (Roecklein et al. 2013; Berman et al. 2018; Laurenzo et al. 2016; Feigl et al. 2018). Moreover, since the studies have used different instruments to assess depression severity, the populations are not directly comparable. The PIPR was similar in 8 young persons with mild-to-moderate depression and 13 healthy controls (Feigl et al. 2018). In another study of 19 persons with unipolar depression of whom the majority were in remission, there was a trend toward a 7% reduction of the PIPR, *p* = 0.07 (Laurenzo et al. 2016). In the largest study to date, 19 in- and outpatients with depression and 20 patients with SAD were assessed with pupillometry during a depressive episode in winter and at follow-up 6 months later (Berman et al. 2018). In both groups with depression, the PIPR was reduced compared with healthy controls. The participants were recruited from a hospital setting which could indicate a more severe illness than in the other studies although their depression scores categorized as mild-to-moderate. In the first pupillometric study in affective disorder performed in persons with SAD, the PIPR was attenuated compared with controls (Roecklein et al. 2013). In SAD, the reduction in ipRGC-responsivity has been suggested as a causal factor, but the association between symptom severity and PIPR remains unresolved in unipolar depression. State marker potential of the PIPR in BD should be evaluated in longitudinal designs of patients across different affective states and preferably with higher symptom severity.

We find a positive association between the PIPR_late_ and illness duration. Due to strong collinearity between age and illness duration, this association was not significant when adjusting for sex and age. As the PIPR is known to be stable across increasing age (Kankipati et al. 2010; Daneault et al. 2016) and since our data do not indicate an association between age and the PIPR_late_ in either the UR or HC groups, we cautiously suggest that the increase in PIPR relates more to illness duration or progression than to higher age. This would be in line with the concept of BD as a progressive disorder presented elsewhere (Kessing and Andersen 2017). A recent OCT study of inpatients with BD did indeed identify a thinning of the retinal nerve fiber layer in BD compared with healthy controls (Khalil et al. 2017). The aforementioned pupillometric studies in unipolar depression have not reported on number of prior episodes or illness duration, hence the association between illness duration and the PIPR constitutes a novel finding. If ipRGC-responsivity is indeed altered in more advanced or severe BD, chronotherapeutic modalities may be more effective in these groups. Blue light-blocking eyewear has been evaluated as an add-on treatment for severe mania in a randomized design in hospitalized patients with a mean illness duration of 20 years (Henriksen et al. 2016). In this population, there was a substantial and rapid reduction in YMRS scores with use of blue-blocking glasses eyewear compared with placebo glasses.

There is a number of limitations to consider in relation to our study. First and foremost, the sample size is modest and do not allow for adjustment for all potential confounding factors. We included only 3 patients with current manic symptoms, why we cannot evaluate any association between hypomania/mania and the PIPR. Importantly, we are also not able to decipher the potential effects of psychotropic medication, since all patients received medication and the majority received combination treatment. By their effects on neurotransmitter systems, psychotropic medications may potentially modify the pupillary motility and/or the sensitivity of the ipRGC-system. A recent study in unmedicated persons found that melatonin suppression was reduced in persons with current but not remitted depression (McGlashan ett al. 2018). No studies have specifically investigated the effects of psychopharmacologic agents on the blue light induced PIPR, but selective serotonin reuptake inhibitors (SSRI) and some antipsychotic agents have been shown to alter the pupil diameter (Koller et al. 2018; Bitsios et al. 1999). An acute dose of the antidepressant citalopram was shown to increase melatonin suppression without affecting the pupillary diameter in a double-blind, placebo-controlled crossover study (McGlashan et al. 2018). No modulatory effects on the PIPR has been reported from post-hoc analyses of antidepressant or anti-manic medications in the referenced pupillometric studies (Roecklein et al. 2013; Berman et al. 2018; Laurenzo et al. 2016; Feigl et al. 2018). Conclusively, medication effects on the pupillary responses or on the circadian system cannot be discarded, although there is no current evidence for drug-related effects on the ipRGC-related pupillary responses, but some evidence for alterations of the resting-state pupillary diameter. We report the PIPR_late_ as percentage pupil constriction relative to baseline in normalized values and not crude numbers, why differences in the resting state pupillary diameters are considered. Another limitation is that we were not able to perform full adjustments for potential effects of photoperiod and time of day due to the sample size. An association between the PIPR and photoperiod has been reported, i.e. a winter-to-summer difference limited to 1–2% (Berman et al. 2018; Laurenzo et al. 2016; Münch et al. 2017) that was primarily related to direct light exposure and not as much to photoperiod (Laurenzo et al. 2016). Our assessments were performed at random dates throughout the year, excluding the winter months, but we made no systematic attempts to match the groups for season or assess light exposure. The main outcome of our study, the comparison of the PIPR_late_ in the 3 groups was adjusted for seasonal but not for diurnal effects. The PIPR undergoes diurnal regulation with an increase in the early morning, a midday plateau and an evening decrease (Münch et al. 2012; Zele et al. 2011). To avoid these morning and evening extremes, we limited the time of assessment to normal office hours, where the PIPR is supposed to display a stable plateau-phase.

Strengths of our study include a thorough diagnostic evaluation to ensure the diagnosis of BD and absence of psychopathology in the two control groups. The inclusion of patients with early BD and their unaffected relatives allows us to specifically assess potential endophenotype features of the ipRGC-responses in BD. We assessed visual acuity and retinal structure to ensure that any pupillometric variation was not related to ocular pathology. Moreover, the implementation of pupillometry in affective disorder research has the potential to add to our understanding of the intertwining of the ipRGC-system and affective regulation. Retinal sensitivity measured during nighttime darkness in melatonin suppression designs may not correspond directly with daytime light sensitivity or ipRGC-function. In glaucoma, where ipRGC degeneration causes attenuations of the PIPR, the correlation between melatonin suppression and the PIPR is rather weak (Munch et al. 2015).

## Conclusions

In this first investigation of the ipRGC-related pupillary responses in BD, we find a reduced ipRGC-response in remitted patients compared with controls, but no signs that such aberrant ipRGC-signaling constitutes an inherited trait feature in BD. The positive association between the ipRGC-responses and illness duration could suggest an alteration of the ipRGC-system over the course of disease. This new hypothesis needs corroboration in longitudinal designs. State marker characteristics of the ipRGC-mediated responses in BD should be tested in larger samples in longitudinal designs and include patients with a higher load of affective symptoms.

## Data Availability

The datasets used during the current study are available from the corresponding author on reasonable request.
